# Supplementary material for: The Physiological and Pathological Role of Acyl-CoA Oxidation
Source: Int J Mol Sci. 2023 Oct 3;24(19):14857. doi: 10.3390/ijms241914857 (PMC10573383; doi:10.3390/ijms241914857)
Supplement: Supplementary file 1 [file ijms-24-14857-s001.zip › ijms-2602879-supplementary.pdf]

Review

Physiological and Pathological Role of Acyl-CoA Oxidation

Sylwia Szrok-Jurga [1,*], Aleksandra Czumaj [2,*], Jacek Turyn [1], Areta Hebanowska [1], Julian Swierczynski [3], Tomasz Sledzinski [2], Ewa Stelmanska [1]

Supplementary Table S1. Sequence variations in fatty acid oxidation genes with clinical significance. Based on: MCID – MalaCards Human Disease Database, OMIM – Online Mendelian Inheritance in Man database, ORPHA – Orphanet database, ACADM – medium-chain acyl-CoA dehydrogenase, ACADS – short-chain acyl-CoA dehydrogenase, ACOX1 - peroxisomes acyl-CoA oxidase 1, ACSL – long-chain fatty acid acyl-CoA synthetase, ACSS – short-chain fatty acyl-CoA synthetases, CPT – carnitine palmitoyltransferase, ECHS1 – short-chain enoyl-CoA hydratase, EHHADH – enoyl-CoA hydratase and 3-hydroxyacyl CoA dehydrogenase, HADH – 3-hydroxyacyl-CoA dehydrogenase.

Gene	Clinical condition	Variations	Inheritance	Prevalence	Severity	Ref.
ABCD1	X linked adrenoleukodystrophy	Single base mutation, deletion, duplication	X-linked recessive	1:17 000	Severe morbidity and mortality in most affected subjects	MCID: ADR007, OMIM:#300100, ORPHA: 43
ACOX1	Peroxisomal acyl-Coa oxidase deficiency (pseudo-NALD)	Single base mutation, deletion	Autosomal recessive	<1/1 000 000	Most children do not survive past early childhood	MCID: PRX028, OMIM: #264470
ACSL4	X-linked non-syndromic intellectual disability	Single base mutation	X-linked recessive	Reported in less that 5 families worldwide	From mild to severe mental retardation	MCID: NNS032, OMIM: #300387,
ACSS2	Orofacial Cleft 1	Single base mutation	Autosomal dominant	Prevalence not determined	Wide phenotypic spectrum	MCID: ORF048, OMIM: #119530
CPT1A	Carnitine palmitoyl-transferase 1 deficiency	Single base mutation, deletion, duplication	Autosomal recessive	<1/1 000 000	Good prognosis with treatment (a low-fat diet, medium-chain TAG supplementation, and avoidance of fasting)	ORPHA: 156, OMIM: #255120, MCID: CRN295
CPT1C	Spastic paraplegia 73	Single base mutation, deletion	Autosomal dominant	Reported in less that 5 families worldwide	Onset and clinical course slowly progressive	ORPHA: 4444099, OMIM: #616282, MCID: SPS160
CPT2	Carnitine palmitoyl-transferase 2 deficiency	Single base mutation, deletion, duplication	Autosomal recessive	<1/1 000 000	Three primary clinical forms: a) neonatal – almost always lethal, b)	ORPHA: 157, OMIM: #608836, #255110,

					infantile – may lead to sudden death, c) myopathic – good prognosis	#600649, MCID: CRN296, CRN294, CRN302
ACADM	Medium-chain acyl-CoA dehydrogenase deficiency	Single base mutation, deletion, indel, duplication, insertion	Autosomal recessive	1/4 000 (Europe), 1/15 000 (Worldwide)	Potentially fatal, normal life expectancy with early diagnosis	ORPHA:42 OMIM: #231680 MCID: MDM001
	Reye syndrome	Single base mutation	Unknown	<1/1 000 000 (Worldwide)	~30% chance of death, ~30% of long-term disability	[1,2]
ACADS	Short-chain acyl-CoA dehydrogenase deficiency	Single base mutation, deletion, indel, duplication	Autosomal recessive	1/35 000 to 1/50 000	Highly variable phenotype, ranging from asymptomatic to severe	OMIM: #201470 MCID: ACY005
ACADVL	Very long-chain acyl-CoA dehydrogenase deficiency	Single base mutation, deletion, indel, duplication, insertion	Autosomal recessive	1/30 000 to 1/120 000	Three main clinical forms: a) severe, with early death; b) milder, childhood form; c) mild adult form	OMIM: #201475 MCID: ACY010
ECHS1	Mitochondrial short-chain enoyl-CoA hydratase 1 deficiency	Single base mutation, deletion, duplication	Autosomal recessive	Prevalence not determined	High mortality rate and poor prognosis	OMIM: #616277 MCID: MTC108
	Leigh syndrome	Single base mutation	Autosomal recessive or mitochondrial inheritance	1-9 / 100 000	Death at a few years of age, poor prognosis	ORPHA:506 OMIM: #256000 MCID: LGH007
EHHADH	Fanconi renotubular syndrome type 3	Single base mutation	Autosomal dominant	Prevalence not determined	With treatment, normal physical and neurocognitive development is usually possible	OMIM: #615605, MCID: FNC049
HADH	3-hydroxyacyl-CoA dehydrogenase deficiency	Single base mutation, deletion	Autosomal recessive	Reported in 31 families worldwide	Highly variable phenotype, sudden infant death may occur	OMIM: #231530, MCID: 3HY005

	Familial hyperinsulinemia hypoglycemia type 4	Single base mutation, deletion	Autosomal recessive	<1/1 000 000	Potentially fatal	ORPHA: 71212, OMIM: #309975, MCID: HYP271
HADHA	Long-chain 3-hydroxy acyl-CoA dehydrogenase deficiency	Single base mutation, deletion, duplication	Autosomal recessive	1/250 000 (Worldwide), 1/120 000 (Poland)	Episodic illness	ORPHA:5, OMIM: #609016, MCID: LNG073
HADHA *HADHB*	Mitochondrial trifunctional protein deficiency	Single base mutation, duplication	Autosomal recessive	1-9/100 000 (Europe)	Three major clinical forms: a) neonatal – severe, lethal condition, b) infantile, c) childhood/adolescent – protracted, with myopathy and neuropathy	ORPHA: 746, OMIM: #609015, MCID: MTC027

Supplementary Table S2 Traits associated with genetic variants of fatty acid oxidation enzymes. CAD – coronary artery disease, CKD – chronic kidney disease, FA – fatty acid, DHA – docosahexaenoic acid, HDL – high-density lipoprotein, LDL – low-density lipoprotein, MUFA – monounsaturated fatty acids, PUFA – polyunsaturated fatty acids, SHBG – sex hormone-binding globulin, TAG – triglyceride, VLDL - very-low-density lipoprotein.

**Enzyme**	**Gene**	**SNP variant**	**Associated trait**	**Nature of change**	**Tested population**	**Ref.**
Long-chain acyl-CoA synthetase	*ACSL1*	rs4862423-T	Fasting glucose	Increased level of glucose	European	[3]
		rs55691245-G, rs72695645-G, rs1996546-G	Type 2 diabetes	Increased risk of diabetes type 2	European, African American, Asian	[4,5]
		rs735949-C	Type 2 diabetes	Decreased risk of diabetes type 2	European	[6]
	ACSL3	rs2461751-G	Electrocardiographic conduction measures	Increased PR interval	Oceanian	[7]
	ACSL4	rs190431955-A	Mean platelet volume	Decreased volume of platelet	European	[8]
	ACSL5	rs58854276-A	Amyotrophic lateral sclerosis	Increased risk of disease	European, East Asian	[9]
		rs58854276-G	HDL cholesterol level	Increased level of blood HDL cholesterol	East Asian	[10]
		rs3736946-G	TAG level	Decreased level of blood TAG	European, African, South Asian	[11]
		rs146605626-A, rs561263417-T, rs146605626-A	Mean platelet volume	Increased volume of platelet	European	[8,12]
		rs2419626-A, rs79671623-A	Platelet count	Decreased number of platelet	European	[8,13]

	ACSL6	rs253942-T	TAG level	Increased level of blood TAG	European	[14]
Medium-chain acyl-CoA synthetase	*ACSM2A*	rs757002-A	Estimated glomerular filtration rate	Decreased rate of glomerular filtration	East Asian Latin American, African American, European	[15]
		rs10163426-T	Indoleacetoylcarnitine, indoleacetylglutamine levels	Increased level of blood indoleacetoylcarnitine and indoleacetylglutamine	European	[16]
		rs6497490-G	Indolepropionate level	Increased level of blood indolepropionate	European	[17]
		rs4783532-G, rs1394678-T	Indolepropionate level	Decreased level of blood indolepropionate	European	[17,18]
	ACSM2B	rs35623745-G	Creatinine level	Decreased level of blood creatinine	European, African, South Asian	[11]
		rs35243287-G	Serum uric acid level	Decreased level of serum uric acid	East Asian	[19]
		rs7499271-T	Serum picolinate level	Decreased level of serum picolinate	Latin American	[20]
		rs62035059-T	Serum picolinate level	Increased level of serum picolinate	Latin American	[20]
		rs142593364-T	Serum β-hydroxyisovalerate level	Increased level of serum β-hydroxyisovalerate	Latin American	[20]
		rs7499271-A, rs977186117-A	Serum phenylacetate level	Increased level of serum phenylacetate	European, African American	[16,21]

		rs11645661-A	3-indolepropionic acid level in CAD	Decreased level in plasma of patients with CAD	East Asian	[22]
		rs73530508-A	L-tryptophan to 3-indolepropionic acid, L-histidine to 3-indolepropionic acid ratio in patients with CAD	Decreased ratio of: L-tryptophan to 3-indolepropionic acid, and L-histidine to 3-indolepropionic acid in plasma of patients with CAD	East Asian	[22]
		rs73530508-A	3-Indolepropionic acid to L-lysine, 3-indolepropionic acid to uridine, 3-indolepropionic acid to adenine, 3-indolepropionic acid to adenine ratio in CAD	Increased ratio of: 3-Indolepropionic acid to L-lysine, 3-indolepropionic acid to uridine, 3-indolepropionic acid to adenine, 3-indolepropionic acid to adenine in plasma of patients with CAD	East Asian	[22]
	ACSM3	rs55792109-T	Creatinine, urate levels	Increased levels of blood creatinine and urate	European, African, South Asian	[11]
		rs55792109-T	Estimated glomerular filtration rate	Decreased glomerular filtration rate	European, African, South Asian	[11]
		rs978161-T	4-hydroxy-phenylacetylglutamine level	Increased level of blood 4-hydroxy-phenylacetylglutamine	European	[16]
	ACSM5	rs142481473-A	Estimated glomerular filtration rate	Increased glomerular filtration rate	European, East Asian, African, Latin American	[23]

					African American, Central Asian, South Asian, Native American	
		rs34927101-T, rs11647589-A	3-phenylpropionate level	Increased level of plasma/serum 3-phenylpropionate	European	[17,18]
		rs9929808-T, rs6497488-T	3-indolepropionic acid level in CAD	Decreased level of plasma 3-indolepropionic acid in patients with CAD	East Asian	[22]
	ACSM6	rs685607-A	Urinary dimethylmalonic acid level in CKD	Increased level of urinary dimethylmalonic acid in patients with CKD	European	[24]
		rs147096448-A	3-hydroxy-2-ethylpropionate level	Decreased level of plasma 3-hydroxy-2-ethylpropionate	European	[17]
Short-chain acyl-CoA synthetase	*ACSS1*	rs4815364-A	Alcohol consumption, bitter alcoholic beverage consumption	Increased number of drinks per week, Increased consumption	European	[25,26]
		rs77671253-G	Blood protein level	Decreased level of blood protein	European	[27]
		rs910527-C, rs76659077-C	Decreased level of cystatin-F	Decreased level of blood cystatin-F	Middle Eastern	[28]
	ACSS2	rs67719508-C	Appendicular lean mass	Increased appendicular lean mass	European	[29]
		rs8123210-G	Acetate level	Decreased level of blood acetate	European	[30]
		rs6088638-C	Hip circumference	Increased hip circumference	European	[31]

	ACSS3	rs11114664-T	Serum γ-glutamyl transferase	Decreased level of serum γ-glutamyl transferase	European, East Asian	[19]
		rs12296937-G	Age-related cataracts	Decreased age at onset	European, African unspecified,	[32]
		rs10862220-T	Walking pace	Decreased walking pace	European	[33]
		rs11114787-T	Alcohol consumption	Increased alcohol consumption	European	[34]
		rs10506274-T, rs12826108-A, rs11835638-C, rs61934664-A	Alcohol consumption	Decreased alcohol consumption	European, East Asian, Latin American, African	[25,35,36]
		rs7138951-G	Plasma amyloid beta peptide concentrations (ABx-42)	Increased concentration of plasma amyloid beta peptide (ABx-42)	European	[37]
Carnitine palmitoyltransferase 1	*CPT1A*	rs2278907-A	Bone density	Increased bone density	European	[38]
		rs11228377-T, rs597539-G	TAG level	Increased blood TAG level	European, East Asian	[19,39]
		rs78863347-T	Urate concentration	Increased serum urate concentration	East Asian	[40]
		rs2229738-T	Serum alkaline phosphatase level	Increased serum alkaline phosphatase level	European, East Asian	[19]
		rs2229738-C	Free cholesterol to total lipids ratio in LDL cholesterol, cholesterol to total lipids in VLDL, PUFA to MUFA ratio, PUFA to total FA ratio, ω-3	Increased ratio of free cholesterol to total lipids ratio in LDL cholesterol, cholesterol to total lipids in VLDL, PUFA to MUFA ratio, PUFA to total FA	European	[30]

			PUFA to total FA ratio, DHA to total FA ratio	ratio, ω-3 PUFA to total FA ratio, DHA to total FA ratio		
			MUFA to total FA, ω-6 PUFA to n-3 PUFA ratio	Decreased ratio of blood MUFA to total FA, ω-6 PUFA to ω-3 PUFA	European	[30]
			n-3 PUFA level	Increased level of blood n-3 PUFA	European	[30,41]
		rs11228374-A, rs2003892-A	HDL cholesterol level	Decreased blood HDL cholesterol level	European, East Asian	[14,19]
		rs11605837-G	HDL cholesterol level, ApoA1 level	Increased blood HDL cholesterol and ApoA1 level.	European	[39]
	CPT1C	rs147472287-T	Lung function	Decreased forced expiratory volume (FEV) to forced vital capacity (FCV) ratio	African	[42]
		rs45617640-T	Serum albumin level, serum total protein level	Decreased level of serum albumin and seru total protein	European, African, South Asian	[11]
Carnitine palmitoyltransferase 2	*CPT2*	rs77466051-A, rs2229291-T, rs1799822-A	Glutarylcarnitine level	Increased level of blood glutarylcarnitine	East Asian, Latin American	[20,43]
		rs1799822-G	Glutarylcarnitine level	Decreased level of blood glutarylcarnitine	European	[16]
		rs11581518-A	Adipoylcarnitine level	Decreased level of blood adipoylcarnitine	Latin American	[20]
Medium-chain acyl-CoA dehydrogenase	*ACADM* (*ACAD1*)	rs61799988-A	hexanoyl-carnitine, octanoyl-carnitine levels	Decreased level of blood hexanoyl-carnitine, octanoyl-carnitine	European	[44]

		rs12126607-A rs4646961-A	Glycine level	Increased level of blood glycine	European	[45–47]
		rs1251075-G rs11161430-T	N-octanoylglutamine, hexanoylglycine, and isocaproylglycine levels	Decreased urinary levels of N-octanoylglutamine, hexanoylglycine, and isocaproylglycine in CKD patients	European	[24]
Short-chain acyl-CoA dehydrogenase	*ACADS* (*ACAD3*)	rs9204-A	2-methylsuccinic acid level	Increased level of 2-methylsuccinic acid in plasma of CAD patients	East Asian	[22]
		rs59063082-T	Acylcarnitine level	Decreased level of blood acylcarnitine	East Asian	[43]
		rs575437-T	Ethylmalonate level	Decreased serum level of ethylmalonate	Latin American	[20]
		rs3916-C	Butyrylcarnitine level	Increased level of blood butyrylcarnitine	European	[46]
			Methylsuccinate level	Increased urinary level of methylsuccinate in CKD patients	European	[24]
		rs34491494-C	Butyrylcarnitine level	Decreased serum level of butyrylcarnitine	Latin American	[20]
		rs2014355-C	Butyrylcarnitine, isobutyrylcarnitine levels	Increased serum levels of butyrylcarnitine and isobutyrylcarnitine in CKD patients	European	[48]
		rs1799958-A	Carnitine, butyrylcarnitine, and isobutyrylcarnitine levels	Decreased serum levels of carnitine, butyrylcarnitine,	European	[48]

				and isobutyrylcarnitine in CKD patients		
			5-hydroxyhexanoate, ethylmalonate, N-formylmethionine, and triacetate lactone levels	Increased urinary levels of 5-hydroxyhexanoate, ethylmalonate, N-formylmethionine, and triacetate lactone in CKD patients	European	[24]
		rs1800556-C	Ethylmalonate level	Decreased serum level of ethylmalonate	Latin American	[20]
Long-chain acyl-CoA dehydrogenase	*ACADL* (*LCAD*, *ACAD4*)	rs2286963-T	Nonaylcarnitine level	Increased serum level of blood nonaylcarnitine	European	[49]
		rs3764913-C	Nonaylcarnitine level in CKD	Increased serum level of nonaylcarnitine level in CKD patients	European, African American	[44,48]
		rs2286963-T	Decadienylcarnitine level	Increased serum level of decadienylcarnitine	European	[49]
		rs2286963-G	Glycine conjugate of $C_9H_{16}O_2$ level	Increased urinary level of glycine conjugate of $C_9H_{16}O_2$	European	[24]
Very long-chain acyl-CoA dehydrogenase	ACADVL (VLCAD, LCACD, ACAD6)	rs77680021-A	Apolipoprotein B levels	Decreased blood level of apolipoprotein B	European, African, South Asian	[11]
			Alkaline phosphatase level	Increased serum level of alkaline phosphatase	European, African, South Asian	[11]

			Total cholesterol level	Decreased level of blood total cholesterol	European, African, South Asian	[11]
			LDL level	Decreased level of blood LDL cholesterol	European, African, South Asian	[11]
			Testosterone level	Increased serum level of testosterone (patients of both sexes)	European, African, South Asian	[11]
			SHBG level	Increased serum level of SHBG (patients of both sexes)	European, African, South Asian	[11]
Acyl-CoA dehydrogenase family member 8	*ACAD8*	rs113488591-C	Isobutyrylcarnitine level	Decreased serum level of isobutyrylcarnitine	Latin American	[20]
		rs113488591-G	Butyrylcarnitine level	Increased serum level of butyrylcarnitine	European	[46]
Acyl-CoA dehydrogenase family member 9	*ACAD9*	rs184937941-T	Blood-cell ratios	Increased lymphocyte-to-monocyte ratio	European	[50]
Acyl-CoA dehydrogenase family member 10	*ACAD10*	rs847888-A	Diastolic blood pressure	Increased diastolic blood pressure	African American, Latin American, European,	[51]
			White blood cell count	Increased lymphocyte count	Latin American	[52]
		rs6490294-A	Platelet count	Decreased cells/L number	African American	[53]
		rs11066015-A	Atrial fibrillation	Decreased atrial flutter	European, East Asian	[19]

			Urea nitrogen	Increased blood level of urea nitrogen	European, East Asian	[19]
			Colon polyp	Decreased risk of polyp of colon	European, East Asian	[19]
			Fish consumption measurement	Decreased fish intake frequency	East Asian	[54]
			HDL cholesterol level	Decreased level of blood HDL cholesterol	East Asian, European	[19]
			LDL cholesterol level	Increased level of blood LDL cholesterol	East Asian, European	[19]
			Body height	Decreased body height	East Asian	[19]
			Mean corpuscular hemoglobin	Decreased mean corpuscular hemoglobin volume	East Asian, European	[19]
			Pulse pressure	Decreased pulse pressure	East Asian, European	[19]
			Uric acid	Decreased blood uric acid level	East Asian	[55,56]
		rs11066008-G	Blood molybdenum	Increased blood molybdenum level	East Asian	[57]
		rs11066008-A	Red blood cell	Decreased red blood cell count	East Asian	[13]
Enoyl-CoA hydratase 1	*ECH1*	rs2229259-T	ECH1 protein level	Increased blood level of ECH1 protein	European	[58]
		rs4802890-G	ECH1 protein level	Decreased blood level of ECH1 protein	European	[27]
Short-chain enoyl-CoA hydratase 1	*ECHS1*	rs140410716-T	Response to aspirin and clopidogrel treatment	Major adverse cardiovascular events in	East Asian	[59]

				response to drugs in acute coronary syndrome		
		rs79000481-C	Acisoga level	Increased serum level	Latin American	[20]
Enoyl-CoA hydratase and 3-hydroxyacyl CoA dehydrogenase	*EHHADH*	rs11322724-T	SHBG level	Increased level of blood SHBG (patients of both sexes)	European, African, South Asian	[11]
		rs6786798-A	Insomnia	Decreased risk	European	[60]
Enoyl-CoA hydratase domain containing 2	*ECHDC2*	rs140559632-C	Total cholesterol, non-HDL cholesterol, and LDL cholesterol	Decreased levels of blood: total cholesterol, non-HDL cholesterol, and LDL cholesterol	European	[14]

Supplementary Table S3. List of abbreviations used in the paper

Abbreviation	Meaning
βOX	β-oxidation
ABC transporter	ATP binding cassette transporter
AcAc	Acetoacetate
ACAD9	Acyl-CoA dehydrogenase DH-9
ACBP	Acyl-CoA-binding protein
ACC	Acetyl-CoA carboxylase
ACS	Acyl-CoA synthetase
ACSL	Long-chain acyl-CoA synthetase
ACSM	Medium-chain acyl-CoA synthetase
ACSS	Short-chain acyl-CoA synthetase
ACOT	Acyl-CoA diesterases
ACOX	Acyl-CoA oxidase
ACSVL	Very long-chain acyl-CoA synthetase
AD	Acyl-CoA dehydrogenase
AR	Androgen receptor
BAT	Brown adipose tissue
BHB	D-β-hydroxybutyrate
BDH	D-β-hydroxybutyrate dehydrogenase
CAC	Acylcarnitine translocase
CD36	Fatty acid translocase (cluster of differentiation 36)
CKD	Chronic kidney disease
CPS	Carbamoyl phosphate synthetase
CPT	Carnitine palmitoyltransferase
CoA	Coenzyme Q
CYP	Cytochrome P-450
DBD	DNA-binding domain
EC	Endothelial cell
ECH	Enoyl-CoA-hydratase
ECHS	Short-chain enoyl-CoA hydratase
ECHS1D	Short-chain enoyl-CoA hydratase 1 deficiency
ECI	3,2-trans-enoyl-CoA isomerase
EHHADH	Enoyl-CoA hydratase and 3-hydroxyacyl CoA dehydrogenase
ER	Endoplasmic reticulum
ESR	Estrogen receptor
ETF	Electron-transferring flavoprotein
FABPc	Cytosolic fatty acid-binding protein
FABPm	Membrane fatty acid-binding protein
FA	Fatty acid
FATP	Fatty acid transporting protein
FAO	Fatty acid oxidation
FAOD	Fatty acid oxidation disorders
FFA	Free fatty acid
GBM	Glioblastoma multiforme
GLUT	Glucose transporter
GSH	Reduced glutathione
HAD	Hydroxy-acyl-CoA dehydrogenase
HADHA	Mitochondrial trifunctional protein, alpha subunit
HADHB	Mitochondrial trifunctional protein, beta subunit
HCC	Hepatocellular carcinoma
HDL	High-density lipoprotein
HF	Heart failure
HMGCL	3-hydroxy-3-methylglutaryl-CoA lyase
HMG-CoA	3-hydroxy-3-methylglutaryl-CoA
HMGCS	3-hydroxy-3-methylglutaryl-CoA synthase
IDH	Isocitrate dehydrogenase

IPF	Idiopathic pulmonary fibrosis
LCAD	Long-chain acyl-CoA dehydrogenase
LCEH	Long-chain enoyl-CoA hydratase
LCHAD	Long-chain hydroxy acyl-CoA dehydrogenase
LCHADD	Long-chain hydroxy acyl-CoA dehydrogenase deficiency
LCKAT	Long-chain fatty acid β-ketothiolase
LCFA	Long-chain fatty acid
LDL	Low-density lipoprotein
MCAD	Medium-chain acyl-CoA dehydrogenase
MCD	Malonyl-CoA decarboxylase
MCFA	Medium-chain fatty acid
MCKAT	Medium-chain ketoacyl-CoA thiolase
ME	Malic enzyme
MTP	Mitochondrial trifunctional protein
MTPD	Mitochondrial trifunctional protein deficiency
NAG	N-acetylglutamate
NATs	N-acetyl transferases
OCTN	Carnitine transporter present in heart, skeletal muscle and kidney
OXPHOS	Oxidative phosphorylation
PDC	Pyruvate dehydrogenase complex
PGC1α	PPARγ coactivator 1α
PKC	Protein kinase C
PPRE	Peroxisome proliferator response element
PPAR	Peroxisome proliferator-activated receptor
pseudo-NALD	Pseudoneonatal adrenoleukodystrophy
PUFA	Polyunsaturated fatty acid
ROS	Reactive oxygen species
RXR	Retinoid X receptor
SCAD	Short-chain acyl-CoA dehydrogenase
SCADD	Short-chain acyl-CoA dehydrogenase deficiency
SCOT	Succinyl-CoA:3-oxoacid-CoA transferase
SCFA	Short-chain fatty acid
SCHAD	Short-chain hydroxy acyl-CoA dehydrogenase
TAG	Triacylglycerol
T2D	Type 2 diabetes
TCA	Tricarboxylic acid / Krebs cycle
TGF	Transforming growth factor
TSPO	Translocator protein
VDAC	Voltage-dependent anion channel
VLCAD	Very long-chain acyl-CoA dehydrogenase
VLDL	Very-low-density lipoprotein
WAT	White adipose tissue
X-ALD	X linked adrenoleukodystrophy

References

1. Pugliese, A.; Beltramo, T.; Torre, D. Reye's and Reye's-like syndromes. *Cell Biochem. Funct.* **2008**, *26*, 741–746, doi:10.1002/CBF.1465.
2. Schrör, K. Aspirin and Reye syndrome: A review of the evidence. *Pediatr. Drugs* 2007, *9*, 195–204.
3. Chen, J.; Spracklen, C.N.; Marenne, G.; Varshney, A.; Corbin, L.J.; Luan, J.; Willems, S.M.; Wu, Y.; Zhang, X.; Horikoshi, M.; et al. The Trans-Ancestral Genomic Architecture of Glycemic Traits. *Nat. Genet.* **2021**, *53*, 840, doi:10.1038/S41588-021-00852-9.
4. Vujkovic, M.; Keaton, J.M.; Lynch, J.A.; Miller, D.R.; Zhou, J.; Tcheandjieu, C.; Huffman, J.E.; Assimes, T.L.; Lorenz, K.; Zhu, X.; et al. Discovery of 318 new risk loci for type 2 diabetes and related vascular outcomes among 1.4 million participants in a multi-ancestry meta-analysis. *Nat. Genet.* **2020**, *52*, 680–691, doi:10.1038/s41588-020-0637-y.
5. Mahajan, A.; Spracklen, C.N.; Zhang, W.; Ng, M.C.Y.; Petty, L.E.; Kitajima, H.; Yu, G.Z.; Rüeger, S.; Speidel, L.; Kim, Y.J.; et al. Multi-ancestry genetic study of type 2 diabetes highlights the power of diverse populations for discovery and translation. *Nat. Genet. 2022 545* **2022**, *54*, 560–572, doi:10.1038/s41588-022-01058-3.
6. Xue, A.; Wu, Y.; Zhu, Z.; Zhang, F.; Kemper, K.E.; Zheng, Z.; Yengo, L.; Lloyd-Jones, L.R.; Sidorenko, J.; Wu, Y.; et al. Genome-wide association analyses identify 143 risk variants and putative regulatory mechanisms for type 2 diabetes. *Nat. Commun.* **2018**, *9*, doi:10.1038/s41467-018-04951-w.
7. Smith, J.G.; Lowe, J.K.; Kovvali, S.; Maller, J.B.; Salit, J.; Daly, M.J.; Stoffel, M.; Altshuler, D.M.; Friedman, J.M.; Breslow, J.L.; et al. Genome-wide association study of electrocardiographic conduction measures in an isolated founder population: Kosrae. *Hear. Rhythm* **2009**, *6*, 634–641, doi:10.1016/j.hrthm.2009.02.022.
8. Vuckovic, D.; Bao, E.L.; Akbari, P.; Lareau, C.A.; Mousas, A.; Jiang, T.; Chen, M.H.; Raffield, L.M.; Tardaguila, M.; Huffman, J.E.; et al. The Polygenic and Monogenic Basis of Blood Traits and Diseases. *Cell* **2020**, *182*, 1214-1231.e11, doi:10.1016/j.cell.2020.08.008.
9. van Rheenen, W.; van der Spek, R.A.A.; Bakker, M.K.; van Vugt, J.J.F.A.; Hop, P.J.; Zwamborn, R.A.J.; de Klein, N.; Westra, H.J.; Bakker, O.B.; Deelen, P.; et al. Common and rare variant association analyses in amyotrophic lateral sclerosis identify 15 risk loci with distinct genetic architectures and neuron-specific biology. *Nat. Genet.* **2021**, *53*, 1636–1648, doi:10.1038/s41588-021-00973-1.
10. Lee, S.B.; Choi, J.E.; Park, B.; Cha, M.Y.; Hong, K.W.; Jung, D.H. Dyslipidaemia—Genotype Interactions with Nutrient Intake and Cerebro-Cardiovascular Disease. *Biomedicines* **2022**, *10*, doi:10.3390/biomedicines10071615.
11. Sinnott-Armstrong, N.; Tanigawa, Y.; Amar, D.; Mars, N.; Benner, C.; Aguirre, M.; Venkataraman, G.R.; Wainberg, M.; Ollila, H.M.; Kiiskinen, T.; et al. Genetics of 35 blood and urine biomarkers in the UK Biobank. *Nat. Genet.* **2021**, *53*, 185–194, doi:10.1038/s41588-020-00757-z.
12. Astle, W.J.; Elding, H.; Jiang, T.; Allen, D.; Ruklisa, D.; Mann, A.L.; Mead, D.; Bouman, H.; Riveros-Mckay, F.; Kostadima, M.A.; et al. The Allelic Landscape of Human Blood Cell Trait Variation and Links to Common Complex Disease. *Cell* **2016**, *167*, 1415-1429.e19, doi:10.1016/j.cell.2016.10.042.
13. Chen, M.H.; Raffield, L.M.; Mousas, A.; Sakaue, S.; Huffman, J.E.; Moscati, A.; Trivedi, B.; Jiang, T.; Akbari, P.; Vuckovic, D.; et al. Trans-ethnic and Ancestry-Specific Blood-Cell Genetics in 746,667 Individuals from 5 Global Populations. *Cell* **2020**, *182*, 1198-1213.e14, doi:10.1016/j.cell.2020.06.045.
14. Graham, S.E.; Clarke, S.L.; Wu, K.H.H.; Kanoni, S.; Zajac, G.J.M.; Ramdas, S.; Surakka, I.; Ntalla, I.; Vedantam, S.; Winkler, T.W.; et al. The power of genetic diversity in genome-wide association studies of lipids. *Nature* **2021**, *600*, 675–679, doi:10.1038/S41586-021-04064-3.
15. Morris, A.P.; Le, T.H.; Wu, H.; Akbarov, A.; van der Most, P.J.; Hemani, G.; Smith, G.D.; Mahajan, A.; Gaulton, K.J.; Nadkarni, G.N.; et al. Trans-ethnic kidney function association study reveals putative causal genes and effects on kidney-specific disease aetiologies. *Nat. Commun.* **2019**, *10*, doi:10.1038/s41467-018-07867-7.

16. Yin, X.; Chan, L.S.; Bose, D.; Jackson, A.U.; VandeHaar, P.; Locke, A.E.; Fuchsberger, C.; Stringham, H.M.; Welch, R.; Yu, K.; et al. Genome-wide association studies of metabolites in Finnish men identify disease-relevant loci. *Nat. Commun.* **2022**, *13*, 19, doi:10.1038/s41467-022-29143-5.
17. Hysi, P.G.; Mangino, M.; Christofidou, P.; Falchi, M.; Karoly, E.D.; Mohney, R.P.; Valdes, A.M.; Spector, T.D.; Menni, C. Metabolome Genome-Wide Association Study Identifies 74 Novel Genomic Regions Influencing Plasma Metabolites Levels. *Metabolites* **2022**, *12*, doi:10.3390/metabo12010061.
18. Shin, S.Y.; Fauman, E.B.; Petersen, A.K.; Krumsiek, J.; Santos, R.; Huang, J.; Arnold, M.; Erte, I.; Forgetta, V.; Yang, T.P.; et al. An atlas of genetic influences on human blood metabolites. *Nat. Genet.* **2014**, *46*, 543–550, doi:10.1038/ng.2982.
19. Sakaue, S.; Kanai, M.; Tanigawa, Y.; Karjalainen, J.; Kurki, M.; Koshiba, S.; Narita, A.; Konuma, T.; Yamamoto, K.; Akiyama, M.; et al. A cross-population atlas of genetic associations for 220 human phenotypes. *Nat. Genet.* **2021**, *53*, 1415–1424, doi:10.1038/S41588-021-00931-X.
20. Feofanova, E. V.; Chen, H.; Dai, Y.; Jia, P.; Grove, M.L.; Morrison, A.C.; Qi, Q.; Daviglus, M.; Cai, J.; North, K.E.; et al. A Genome-wide Association Study Discovers 46 Loci of the Human Metabolome in the Hispanic Community Health Study/Study of Latinos. *Am. J. Hum. Genet.* **2020**, *107*, 849–863, doi:10.1016/j.ajhg.2020.09.003.
21. Yu, B.; Zheng, Y.; Alexander, D.; Morrison, A.C.; Coresh, J.; Boerwinkle, E. Genetic Determinants Influencing Human Serum Metabolome among African Americans. *PLoS Genet.* **2014**, *10*, doi:10.1371/journal.pgen.1004212.
22. Wang, Z.; Zhu, Q.; Liu, Y.; Chen, S.; Zhang, Y.; Ma, Q.; Chen, X.; Liu, C.; Lei, H.; Chen, H.; et al. Genome-wide association study of metabolites in patients with coronary artery disease identified novel metabolite quantitative trait loci. *Clin. Transl. Med.* **2021**, *11*, doi:10.1002/CTM2.290.
23. Liu, H.; Doke, T.; Guo, D.; Sheng, X.; Ma, Z.; Park, J.; Vy, H.M.T.; Nadkarni, G.N.; Abedini, A.; Miao, Z.; et al. Epigenomic and transcriptomic analyses define core cell types, genes and targetable mechanisms for kidney disease. *Nat. Genet.* **2022**, *54*, 950–962, doi:10.1038/S41588-022-01097-W.
24. Schlosser, P.; Li, Y.; Sekula, P.; Raffler, J.; Grundner-Culemann, F.; Pietzner, M.; Cheng, Y.; Wuttke, M.; Steinbrenner, I.; Schultheiss, U.T.; et al. Genetic studies of urinary metabolites illuminate mechanisms of detoxification and excretion in humans. *Nat. Genet. 2020 522* **2020**, *52*, 167–176, doi:10.1038/s41588-019-0567-8.
25. Liu, M.; Jiang, Y.; Wedow, R.; Li, Y.; Brazel, D.M.; Chen, F.; Datta, G.; Davila-Velderrain, J.; McGuire, D.; Tian, C.; et al. Association studies of up to 1.2 million individuals yield new insights into the genetic etiology of tobacco and alcohol use. *Nat. Genet.* **2019**, *51*, 237–244, doi:10.1038/s41588-018-0307-5.
26. Karlsson Linnér, R.; Biroli, P.; Kong, E.; Meddens, S.F.W.; Wedow, R.; Fontana, M.A.; Lebreton, M.; Tino, S.P.; Abdellaoui, A.; Hammerschlag, A.R.; et al. Genome-wide association analyses of risk tolerance and risky behaviors in over 1 million individuals identify hundreds of loci and shared genetic influences. *Nat. Genet. 2019 512* **2019**, *51*, 245–257, doi:10.1038/s41588-018-0309-3.
27. Emilsson, V.; Ilkov, M.; Lamb, J.R.; Finkel, N.; Gudmundsson, E.F.; Pitts, R.; Hoover, H.; Gudmundsdottir, V.; Horman, S.R.; Aspelund, T.; et al. Co-regulatory networks of human serum proteins link genetics to disease. *Science* **2018**, *361*, doi:10.1126/SCIENCE.AAQ1327.
28. Thareja, G.; Belkadi, A.; Arnold, M.; Albagha, O.M.E.; Graumann, J.; Schmidt, F.; Grallert, H.; Peters, A.; Gieger, C.; Consortium, T.Q.G.P.R.; et al. Differences and commonalities in the genetic architecture of protein quantitative trait loci in European and Arab populations. *Hum. Mol. Genet.* **2022**, *00*, 1–10, doi:10.1093/hmg/ddac243.
29. Hernandez Cordero, A.I.; Gonzales, N.M.; Parker, C.C.; Sokolof, G.; Vandenbergh, D.J.; Cheng, R.; Abney, M.; Sko, A.; Douglas, A.; Palmer, A.A.; et al. Genome-wide Associations Reveal Human-Mouse Genetic Convergence and Modifiers of Myogenesis, CPNE1 and STC2. *Am. J. Hum. Genet.* **2019**, *105*, 1222–1236, doi:10.1016/j.ajhg.2019.10.014.
30. Richardson, T.G.; Leyden, G.M.; Wang, Q.; Bell, J.A.; Elsworth, B.; Smith, G.D.; Holmes, M. V. Characterising metabolomic signatures of lipid-modifying therapies through drug target mendelian randomisation. *PLoS Biol.* **2022**, *20*,

doi:10.1371/journal.pbio.3001547.
31. Christakoudi, S.; Evangelou, E.; Riboli, E.; Tsilidis, K.K. GWAS of allometric body-shape indices in UK Biobank identifies loci suggesting associations with morphogenesis, organogenesis, adrenal cell renewal and cancer. *Sci. Rep.* **2021**, *11*, 10688, doi:10.1038/s41598-021-89176-6.
32. Ritchie, M.D.; Verma, S.S.; Hall, M.A.; Goodloe, R.J.; Berg, R.L.; Carrell, D.S.; Carlson, C.S.; Chen, L.; Crosslin, D.R.; Denny, J.C.; et al. Electronic medical records and genomics (eMERGE) network exploration in cataract: Several new potential susceptibility loci. *Mol. Vis.* **2014**, *20*, 1281–1295.
33. Timmins, I.R.; Zaccardi, F.; Nelson, C.P.; Franks, P.; Yates, T.; Dudbridge, F. Genome-wide association study of self-reported walking pace suggests beneficial effects of brisk walking on health and survival. *Commun. Biol.* **2020**, *3*, doi:10.1038/s42003-020-01357-7.
34. Evangelou, E.; Gao, H.; Chu, C.; Ntritsos, G.; Blakeley, P.; Butts, A.R.; Pazoki, R.; Suzuki, H.; Koskeridis, F.; Yiorkas, A.M.; et al. New alcohol-related genes suggest shared genetic mechanisms with neuropsychiatric disorders. *Nat. Hum. Behav.* **2019**, *3*, 950–961, doi:10.1038/s41562-019-0653-z.
35. Zhou, H.; Sealock, J.M.; Sanchez-Roige, S.; Clarke, T.K.; Levey, D.F.; Cheng, Z.; Li, B.; Polimanti, R.; Kember, R.L.; Smith, R.V.; et al. Genome-wide meta-analysis of problematic alcohol use in 435,563 individuals yields insights into biology and relationships with other traits. *Nat. Neurosci.* **2020**, *23*, 809–818, doi:10.1038/s41593-020-0643-5.
36. Saunders, G.R.B.; Wang, X.; Chen, F.; Jang, S.K.; Liu, M.; Wang, C.; Gao, S.; Jiang, Y.; Khunsriraksakul, C.; Otto, J.M.; et al. Genetic diversity fuels gene discovery for tobacco and alcohol use. *Nature* **2022**, doi:10.1038/s41586-022-05477-4.
37. Chouraki, V.; De Bruijn, R.F.A.G.; Chapuis, J.; Bis, J.C.; Reitz, C.; Schraen, S.; Ibrahim-Verbaas, C.A.; Grenier-Boley, B.; Delay, C.; Rogers, R.; et al. A genome-wide association meta-analysis of plasma Aβ peptides concentrations in the elderly. *Mol. Psychiatry* **2014**, *19*, 1326–1335, doi:10.1038/mp.2013.185.
38. Medina-Gomez, C.; Kemp, J.P.; Trajanoska, K.; Luan, J.; Chesi, A.; Ahluwalia, T.S.; Mook-Kanamori, D.O.; Ham, A.; Hartwig, F.P.; Evans, D.S.; et al. Life-Course Genome-wide Association Study Meta-analysis of Total Body BMD and Assessment of Age-Specific Effects. *Am. J. Hum. Genet.* **2018**, *102*, 88–102, doi:10.1016/j.ajhg.2017.12.005.
39. Richardson, T.G.; Sanderson, E.; Palmerid, T.M.; Korpelaid, M.A.; Ference, B.A.; Smith, G.D.; Holmes, M. V. Evaluating the relationship between circulating lipoprotein lipids and apolipoproteins with risk of coronary heart disease: A multivariable Mendelian randomisation analysis. *PLoS Med.* **2020**, *17*, doi:10.1371/JOURNAL.PMED.1003062.
40. Tin, A.; Marten, J.; Halperin Kuhns, V.L.; Li, Y.; Wuttke, M.; Kirsten, H.; Sieber, K.B.; Qiu, C.; Gorski, M.; Yu, Z.; et al. Target genes, variants, tissues and transcriptional pathways influencing human serum urate levels. *Nat. Genet.* **2019**, *51*, 1459–1474, doi:10.1038/s41588-019-0504-x.
41. Borges, M.C.; Haycock, P.C.; Zheng, J.; Hemani, G.; Holmes, M. V; Davey Smith, G.; Hingorani, A.D.; Lawlor, D.A. Role of circulating polyunsaturated fatty acids on cardiovascular diseases risk: analysis using Mendelian randomization and fatty acid genetic association data from over 114,000 UK Biobank participants. *BMC Med.* **2022**, *20*, doi:10.1186/s12916-022-02399-w.
42. Wyss, A.B.; Sofer, T.; Lee, M.K.; Terzikhan, N.; Nguyen, J.N.; Lahousse, L.; Latourelle, J.C.; Smith, A.V.; Bartz, T.M.; Feitosa, M.F.; et al. Multiethnic meta-analysis identifies ancestry-specific and cross-ancestry loci for pulmonary function. *Nat. Commun.* **2018**, *9*, doi:10.1038/s41467-018-05369-0.
43. Chai, J.F.; Raichur, S.; Khor, I.W.; Torta, F.; Chew, W.S.; Herr, D.R.; Ching, J.; Kovalik, J.P.; Khoo, C.M.; Wenk, M.R.; et al. Associations with metabolites in Chinese suggest new metabolic roles in Alzheimer's and Parkinson's diseases. *Hum. Mol. Genet.* **2020**, *29*, 189–201, doi:10.1093/HMG/DDZ246.
44. Rhee, E.P.; Surapaneni, A.; Zheng, Z.; Zhou, L.; Dutta, D.; Arking, D.E.; Zhang, J.; Duong, T.V.; Chatterjee, N.; Luo, S.; et al. Trans-ethnic genome-wide association study of blood metabolites in the Chronic Renal Insufficiency Cohort (CRIC) study. *Kidney Int.* **2022**, *101*, 814–823, doi:10.1016/j.kint.2022.01.014.

45. Jia, Q.; Han, Y.; Huang, P.; Woodward, N.C.; Gukasyan, J.; Kettunen, J.; Ala-Korpela, M.; Anufrieva, O.; Wang, Q.; Perola, M.; et al. Genetic Determinants of Circulating Glycine Levels and Risk of Coronary Artery Disease. *J. Am. Heart Assoc.* **2019**, *8*, doi:10.1161/JAHA.119.011922.
46. Lotta, L.A.; Pietzner, M.; Stewart, I.D.; Wittemans, L.B.L.; Li, C.; Bonelli, R.; Raffler, J.; Biggs, E.K.; Oliver-Williams, C.; Auyeung, V.P.W.; et al. Cross-platform genetic discovery of small molecule products of metabolism and application to clinical outcomes. *Nat. Genet.* **2021**, *53*, 54, doi:10.1038/S41588-020-00751-5.
47. Wittemans, L.B.L.; Lotta, L.A.; Oliver-Williams, C.; Stewart, I.D.; Surendran, P.; Karthikeyan, S.; Day, F.R.; Koulman, A.; Imamura, F.; Zeng, L.; et al. Assessing the causal association of glycine with risk of cardio-metabolic diseases. *Nat. Commun.* **2019**, *10*, doi:10.1038/S41467-019-08936-1.
48. Li, Y.; Sekula, P.; Wuttke, M.; Wahrheit, J.; Hausknecht, B.; Schultheiss, U.T.; Gronwald, W.; Schlosser, P.; Tucci, S.; Ekici, A.B.; et al. Genome-Wide Association Studies of Metabolites in Patients with CKD Identify Multiple Loci and Illuminate Tubular Transport Mechanisms. *J. Am. Soc. Nephrol.* **2018**, *29*, 1513–1524, doi:10.1681/ASN.2017101099.
49. Illig, T.; Gieger, C.; Zhai, G.; Römisch-Margl, W.; Wang-Sattler, R.; Prehn, C.; Altmaier, E.; Kastenmüller, G.; Kato, B.S.; Mewes, H.W.; et al. A genome-wide perspective of genetic variation in human metabolism. *Nat. Genet.* **2010**, *42*, 137–141, doi:10.1038/NG.507.
50. Kachuri, L.; Jeon, S.; DeWan, A.T.; Metayer, C.; Ma, X.; Witte, J.S.; Chiang, C.W.K.; Wiemels, J.L.; de Smith, A.J. Genetic determinants of blood-cell traits influence susceptibility to childhood acute lymphoblastic leukemia. *Am. J. Hum. Genet.* **2021**, *108*, 1823–1835, doi:10.1016/J.AJHG.2021.08.004.
51. Gouveia, M.H.; Bentley, A.R.; Leonard, H.; Meeks, K.A.C.; Ekoru, K.; Chen, G.; Nalls, M.A.; Simonsick, E.M.; Tarazona-Santos, E.; Lima-Costa, M.F.; et al. Trans-ethnic meta-analysis identifies new loci associated with longitudinal blood pressure traits. *Sci. Rep.* **2021**, *11*, doi:10.1038/S41598-021-83450-3.
52. Hu, Y.; Bien, S.A.; Nishimura, K.K.; Haessler, J.; Hodonsky, C.J.; Baldassari, A.R.; Highland, H.M.; Wang, Z.; Preuss, M.; Sitlani, C.M.; et al. Multi-ethnic genome-wide association analyses of white blood cell and platelet traits in the Population Architecture using Genomics and Epidemiology (PAGE) study. *BMC Genomics* **2021**, *22*, doi:10.1186/S12864-021-07745-5.
53. Qayyum, R.; Snively, B.M.; Ziv, E.; Nalls, M.A.; Liu, Y.; Tang, W.; Yanek, L.R.; Lange, L.; Evans, M.K.; Ganesh, S.; et al. A meta-analysis and genome-wide association study of platelet count and mean platelet volume in African Americans. *PLoS Genet.* **2012**, *8*, doi:10.1371/journal.pgen.1002491.
54. Igarashi, M.; Nogawa, S.; Kawafune, K.; Hachiya, T.; Takahashi, S.; Jia, H.; Saito, K.; Kato, H. Identification of the 12q24 locus associated with fish intake frequency by genome-wide meta-analysis in Japanese populations. *Genes Nutr.* **2019**, *14*, doi:10.1186/S12263-019-0646-6.
55. Cho, S.K.; Kim, B.; Myung, W.; Chang, Y.; Ryu, S.; Kim, H.N.; Kim, H.L.; Kuo, P.H.; Winkler, C.A.; Won, H.H. Polygenic analysis of the effect of common and low-frequency genetic variants on serum uric acid levels in Korean individuals. *Sci. Rep.* **2020**, *10*, doi:10.1038/S41598-020-66064-Z.
56. Yasukochi, Y.; Sakuma, J.; Takeuchi, I.; Kato, K.; Oguri, M.; Fujimaki, T.; Horibe, H.; Yamada, Y. Identification of CDC42BPG as a novel susceptibility locus for hyperuricemia in a Japanese population. *Mol. Genet. Genomics* **2018**, *293*, 371–379, doi:10.1007/S00438-017-1394-1.
57. Yang, W.; Li, L.; Feng, X.; Cheng, H.; Ge, X.; Bao, Y.; Huang, L.; Wang, F.; Liu, C.; Chen, X.; et al. Genome-wide association and Mendelian randomization study of blood copper levels and 213 deep phenotypes in humans. *Commun. Biol.* **2022**, *5*, doi:10.1038/S42003-022-03351-7.
58. Gudjonsson, A.; Gudmundsdottir, V.; Axelsson, G.T.; Gudmundsson, E.F.; Jonsson, B.G.; Launer, L.J.; Lamb, J.R.; Jennings, L.L.; Aspelund, T.; Emilsson, V.; et al. A genome-wide association study of serum proteins reveals shared loci with common diseases. *Nat. Commun.* **2022**, *13*, doi:10.1038/S41467-021-27850-Z.

59. Liu, X.; Xu, H.; Xu, H.; Geng, Q.; Mak, W.H.; Ling, F.; Su, Z.; Yang, F.; Zhang, T.; Chen, J.; et al. New genetic variants associated with major adverse cardiovascular events in patients with acute coronary syndromes and treated with clopidogrel and aspirin. *Pharmacogenomics J.* **2021**, *21*, 664–672, doi:10.1038/S41397-021-00245-5.
60. Watanabe, K.; Jansen, P.R.; Savage, J.E.; Nandakumar, P.; Wang, X.; Agee, M.; Aslibekyan, S.; Auton, A.; Bell, R.K.; Bryc, K.; et al. Genome-wide meta-analysis of insomnia prioritizes genes associated with metabolic and psychiatric pathways. *Nat. Genet.* **2022**, *54*, 1125–1132, doi:10.1038/S41588-022-01124-W.
